# Patient and caregiver experiences with a patient-support program for setmelanotide treatment of patients with Bardet–Biedl syndrome

**DOI:** 10.1186/s13023-025-03835-9

**Published:** 2025-06-08

**Authors:** Ilja Finkelberg, Ioanna M. Polichronidou, Tom Hühne, Pia Brensing, Sinem Karaterzi, Johannes Jaegers, Anja Gäckler, Lars Pape, Metin Cetiner

**Affiliations:** 1 Pediatric Clinic II, Department of Pediatric Nephrology, Gastroenterology, Endocrinology and Sonography, University Hospital Essen, University of Duisburg- Essen, Essen, Germany; 2 Department of Nephrology, University Hospital Essen, University of Duisburg-Essen, Essen, Germany

**Keywords:** Bardet–Biedl syndrome, Patient experience, Caregiver experience, Real-world evidence, Melanocortin-4 receptor, Setmelanotide, Hyperphagia, Hunger, Obesity

## Abstract

**Background:**

Bardet–Biedl syndrome (BBS) is a rare genetic disease caused by impaired cilium function and characterized by a plethora of symptoms, including hyperphagia and early-onset obesity, that negatively affect patient and caregiver quality of life. Here, we assessed real-world patient expectations and experiences before and during treatment with setmelanotide, a melanocortin-4 receptor agonist shown to reduce hunger and body weight in patients with BBS.

**Methods:**

An online survey was conducted to capture the real-world experience of patients with BBS and their caregivers regarding setmelanotide treatment and the use of a specialist nurse support service aimed at educating patients and their caregivers, and enabling them to administer injections independently. The survey was administered between January 2024 and May 2024 to participants who began treatment between June 2023 and December 2023 at a single center in Germany.

**Results:**

Of the 35 respondents, 10 were pediatric patients, 13 were adult patients, and 12 were caregivers. Prior to treatment, the most commonly reported symptoms by pediatric patients and caregivers were insatiable hunger (80% and 83%, respectively) and obesity (50% and 92%, respectively); for adult patients, key symptoms were vision loss (92%) and obesity (69%). Setmelanotide reduced feelings of insatiable hunger and had a positive effect on body weight: ≥ 92% of respondents across survey groups reported feeling less hunger, feeling satiated after meals, and a stable body weight or weight loss (mean BMI z-score ± SD at start: 3.12 ± 0.89, change after 6 months: −0.47 ± 0.37). Improvements in mobility, mood, and behavior were also reported. The specialist nurse support service was rated excellent by all respondents. The personalized approach contributed to high patient and caregiver satisfaction, enabled most of them to administer the drug independently, and ensured high treatment adherence, without any patients discontinuing setmelanotide treatment.

**Conclusion:**

Based on this real-world survey of patients with BBS and their caregivers, setmelanotide improved key symptoms related to insatiable hunger and obesity. Personalized multidimensional nursing support at the start of treatment can help address unmet support needs in BBS and may contribute to high rates of treatment satisfaction and adherence.

**Supplementary Information:**

The online version contains supplementary material available at 10.1186/s13023-025-03835-9.

## Introduction

Bardet–Biedl syndrome (BBS) is a rare, genetically heterogeneous, autosomal recessive disease caused by impaired primary cilium function. Clinically, BBS is associated with retinal degeneration and early loss of vision, hyperphagia, early-onset obesity, polydactyly, and impairments in renal, hepatic, and cognitive function [1–5]. The reported prevalence of BBS ranges from 1:100,000 to 1:160,000 in North America and Europe, but may be higher (1:13,500 to 1:18,000) in some isolated populations [1, 4, 6–8].

Patients with BBS experience significant disease-related health burden that both reduces quality of life (QoL) and negatively affects the lives of caregivers, parents, and other family members [9–11]. Hyperphagia and obesity are among the most distressing manifestations of the disease and constitute a substantial burden for both patients and caregivers [9, 12, 13]. Primary cilia signaling has an influential role in the hypothalamic melanocortin-4 receptor (MC4R) pathway, which is responsible for controlling energy balance, hunger, and satiety; therefore, impaired primary ciliary signaling results in hyperphagia, an insatiable form of hunger, associated with abnormal food-seeking behaviors [14]. This drives the development of obesity, frequently with onset in infancy [5, 9, 13].

Setmelanotide is an MC4R agonist that restores MC4R pathway signaling. Setmelanotide acts on MC4Rs downstream of the locus for the ciliopathy-related impaired melanocortin signaling seen in BBS [15]. In clinical trials, setmelanotide has been shown to reduce hunger and to maintain/reduce body weight in patients with BBS [16–18]. In 2022, setmelanotide became the first treatment approved by the European Medicines Agency for the treatment of obesity and the control of hunger in children (aged ≥ 6 years) and adults with genetically confirmed BBS. It is also approved for use in patients with biallelic proopiomelanocortin (POMC) deficiency (including *PCSK1*) or biallelic leptin receptor (LEPR) deficiency caused by loss-of-function variants. Since July 2024, setmelanotide is approved in these diseases for children aged ≥ 2 years [19, 20].

Patients with BBS who are treated with setmelanotide may benefit from targeted supportive measures, including patient education and multidisciplinary support from psychologists, nutritionists, and nurses [21]. Co-ordination of care for patients with rare diseases is often suboptimal, and patients affected by rare diseases, such as BBS, generally have high unmet support needs [22, 23]. In Germany, a specialist nurse service was established to support patients with BBS and their caregivers at the start of treatment with daily subcutaneous injections of setmelanotide. The aim of this study was to determine real-world patient expectations and experiences with setmelanotide treatment, and how patients and their caregivers experienced and valued the specialist nurse support service.

## Methods

This single-center study involved a one-time survey fielded to patients with BBS who were treated at the University Hospital Essen, Germany, or caregivers of patients (i.e. a parent, grandparent, or older sibling), who were receiving support from the specialist nurse support service. This service provided daily or weekly education and support from a certified nurse specialist regarding setmelanotide injections, with the aim of enabling patients and their caregivers to administer injections independently. In addition, all patients had access to injection training and prescription delivery through this service, as well as support from a patient association.

Participants were asked to complete an anonymous online survey administered between 4 January 2024 and 15 May 2024. Participants with visual impairment were offered a VoiceOver function on their digital device to complete the questionnaire. All patients had initiated setmelanotide therapy for BBS (between June 2023 and December 2023) prior to completion of the survey. The survey was developed based on the methodology used in a similar study assessing perspectives of patients with prurigo nodularis [24]. Three versions of the survey were created: one for pediatric patients with BBS (< 18 years of age), one for adult patients with BBS (≥ 18 years of age), and one for caregivers of pediatric patients who were too young or who could not self-report. The surveys consisted of 17, 20, and 20 questions, respectively. These are shown in Supplementary Table 1, Additional file 1. The surveys were designed to take approximately 20–25 min to complete. Topics covered by the survey were disease symptoms prior to starting treatment; expectations and concerns regarding treatment; changes in disease symptoms noticed after starting treatment; treatment experience; and experience with the specialist nurse support service. Anonymized survey results were analyzed using descriptive statistics and, if applicable, the results are presented as means and percentages.

## Results

### Patient characteristics

A total of 35 participants were included in the study, including 10 pediatric patients with BBS, 13 adult patients with BBS, and 12 caregivers. Baseline characteristics are summarized in Table 1 and Supplementary Table 2, Additional file 1. Pediatric patients had an even gender distribution and a mean age of 12 years (range 6–18). Adult patients were mostly female (62%) and had a mean age of 23 years (range 18–52). All caregivers were parents to children, who had a mean age of 11 years (range 6–18), most of whom were female (67%). The proportion of single parents was 19%, and no grandparents or siblings were involved in direct care in this study cohort. In all three groups, the patients were predominantly German-speaking and of German origin. 46% of patient received their diagnosis of BBS between 0 and 6 years of age, 38% received their diagnosis after the age of 6 years and for 17% of patients, age at diagnosis was not reported.

**Table 1:** Patient characteristics

	Pediatric patients (*n* = 10)	Adult patients (*n* = 13)	Caregivers describing patients (*n* = 12)
**Sex**, *n* (%)			
Male	5 (50)	5 (38)	4 (33)
Female	5 (50)	8 (62)	8 (67)
**Age in years**,** n (%)**			
6–9	3 (30)	0	4 (33)
10–13	5 (50)	0	4 (33)
14–18	2 (20)	0	2 (17)
18–25	0	6 (46)	0
26–30	0	2 (15)	0
30–35	0	1 (8)	0
36–40	0	1 (8)	0
Not specified	0	3 (23)	2 (17)
**Country of origin**,** n (%)**			
Germany	9 (90)	11 (84)	11 (92)
Turkey	1 (10)	0	0
Portugal	0	1 (8)	0
Lebanon	0	1 (8)	1 (8)
**Mother tongue**,** n (%)**			
German	9 (90)	9 (69)	8 (67)
Turkish	1 (10)	2 (15)	1 (8)
Portuguese	0	1 (8)	0
Arabic	0	1 (8)	2 (17)
Kurdish	0	0	1 (8)
**Ethnicity**,** n (%)**			
European/Eurasian	10 (100)	13 (100)	10 (83)
African	0	0	2 (17)

At the time of the survey, all patients were receiving treatment with setmelanotide under care in a specialized center, including pediatric and adult nephrologists and endocrinologists. In addition, in some cases, BBS disease symptoms were supervised and managed in parallel by a general practitioner (15% of adult patients), an ophthalmologist/otolaryngologist (8% of adult patients), or an outpatient pediatrician (33%).

### Impairment due to disease symptoms before starting setmelanotide treatment

Survey participants were asked to recall disease symptoms prior to starting setmelanotide treatment. Insatiable hunger was the most common symptom reported by pediatric patients (80% responded with either 4 or 5 on a 5-point scale, where 1 indicates “does not apply” and 5 indicates “applies to a high degree”), followed by being overweight (50%). For most of the symptoms perceived prior to treatment, a higher percentage of adult patients responded with a 4 or 5 than pediatric patients, and being overweight was reported as a symptom by 69% of adult patients (see Supplementary Fig. 1, Additional file 2). Insatiable hunger in adult patients prior to treatment start could not be reported due to a technical mistake in the survey. The symptoms prior to treatment that were most commonly reported by caregivers were patients being overweight (92%) and having insatiable hunger (83%). It is important to recognize that vision loss was also a significant impairment reported by 92% of adult patients, 75% of caregivers and 30% of pediatric patients (see Supplementary Fig. 1, Additional file 2).

### Expectations and concerns before starting setmelanotide treatment

When asked to recall their expectations prior to starting therapy, most pediatric patients expected setmelanotide therapy to make them less hungry (90%), reduce body weight or become thinner (90%), and improve QoL (90%) (see Supplementary Fig. 2, Additional file 2). 40% of pediatric patients expected treatment to reduce the burden on their family. Adult patients expected setmelanotide treatment to reduce feelings of insatiable hunger (92%), improve QoL (54%), and reduce body weight (77%). Compared with pediatric patients, adult patients had lower expectations for psychosocial improvements (e.g. minimizing family burden, social exclusion, or bullying). Caregivers generally had greater expectations for treatment than pediatric or adult patients, particularly for improvements in cognitive ability (58%) and absenteeism (25%; see Supplementary Fig. 2, Additional file 2).

Key concerns before starting treatment with setmelanotide among pediatric patients included fear of injections (60%), insufficient effectiveness (30%), side effects (20%), and being left alone with the treatment/injection (20%; see Supplementary Fig. 3, Additional file 2). In adult patients, top concerns prior to treatment included side effects (62%), insufficient effectiveness (31%), and difficult injection preparation (31%). Similarly, caregivers reported that their main concerns were related to side effects (67%), injection fears (50%), insufficient effectiveness (50%), difficult drug preparation (42%), and the time required to administer the drug (33%).

### Effects of setmelanotide treatment

All patients reported treatment effects of setmelanotide, mostly within days (pediatric patients 40%; adult patients 54%; caregivers 42%) or weeks of starting therapy (pediatric patients 60%; adult patients 38%; caregivers 58%). 8% of adult patients only noticed effects of setmelanotide treatment after a few months.

**Fig. 1:**
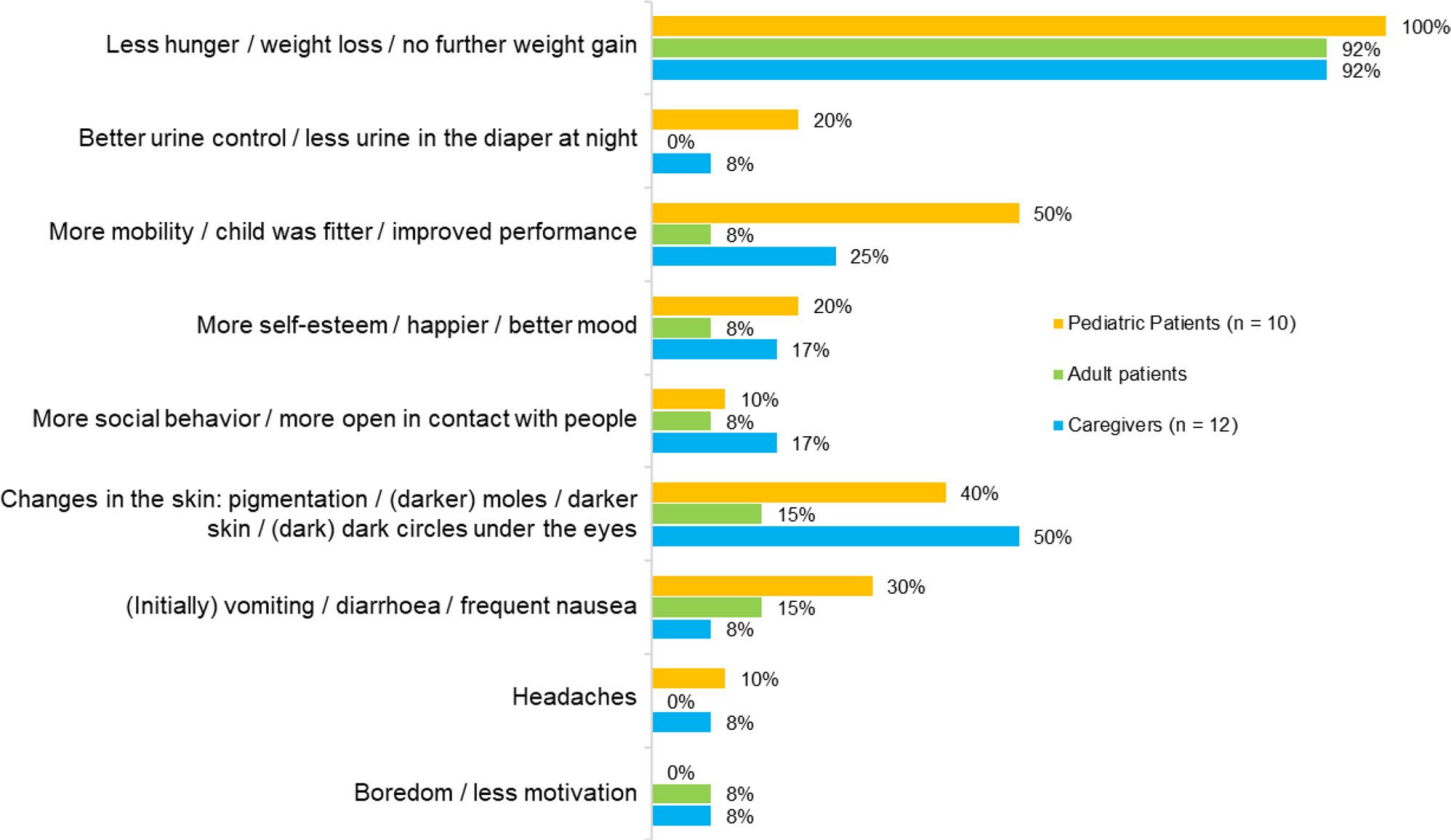
Initial perceived changes after starting setmelanotide therapy* *Changes were noticed within a few days at the earliest, a few weeks at the latest

When asked what the initial perceived changes after starting treatment with setmelanotide were, the survey participants predominantly indicated a reduction in feelings of hunger or weight loss/no further weight gain (Fig. 1). Mean BMI z-score ± SD at the start of setmelanotide treatment was 3.12 ± 0.89, which was reduced by − 0.47 ± 0.37 after 6 months.

**Fig. 2:**
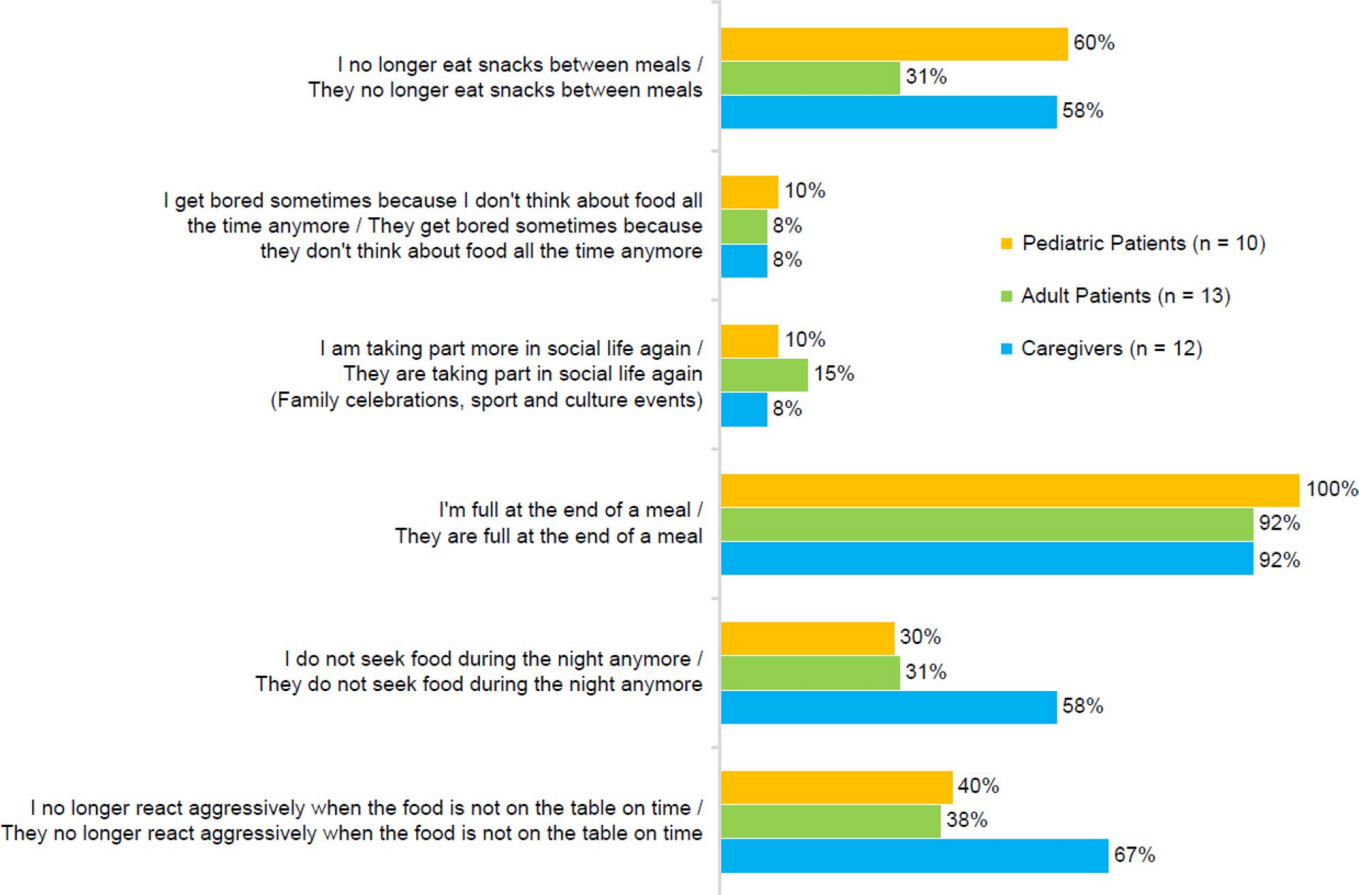
Changes in the feeling of hunger after starting setmelanotide therapy. Proportion of study participants reporting 4 or 5 on a scale where 1 = “does not apply” and 5 = “applies to a high degree”

Most patients (≥ 92% of respondents across all three survey groups) reported feeling satiated after meals. Approximately 60% of pediatric patients and caregivers reported a reduction in snacking between meals, compared with 31% of adult patients. Caregivers were more likely to report reductions in seeking food at night (58% compared with 30% and 31% for pediatric and adult patients, respectively) and acting aggressively when food was not available on time (67% compared with 40% and 38%, respectively) (Fig. 2).

**Fig. 3:**
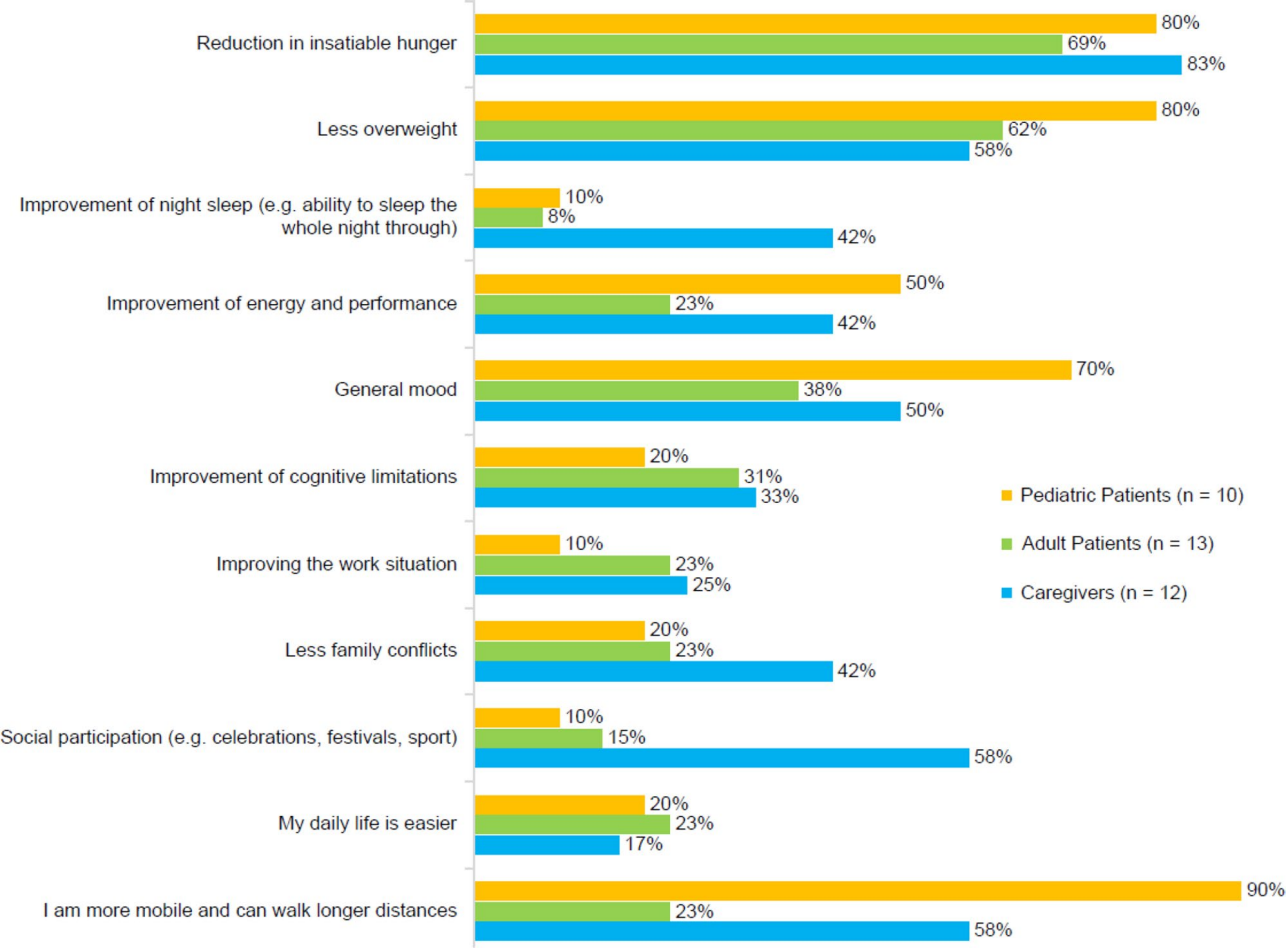
Overall improvements after starting setmelanotide therapy

In pediatric patients, the most reported improvements with setmelanotide therapy were improved mobility (90%), reduced hunger (80%), reduced body weight (80%), and better mood (70%; Fig. 3). In adult patients, the most common improvements were reduced hunger (69%), reduced body weight (62%), and better mood (38%). Adult patients were less likely to report improvements in energy (23% compared with 50% and 42% for pediatric patients and caregivers, respectively) and mobility (23% compared with 90% and 58%, respectively). Caregivers were more likely to report an increase in social participation (58% compared with 10% and 15% of pediatric and adult patients, respectively) and a decrease in family conflicts (42% compared with 20% and 23%, respectively).

Figure 1 also summarizes the adverse events perceived when starting treatment. The most commonly reported change was a change in skin pigmentation (pediatric patients 40%; adult patients 15%; caregivers 50%). Gastrointestinal symptoms, such as nausea, vomiting, and diarrhea, were reported by 30% of pediatric patients but only 8% of caregivers.

Across the three survey groups, most respondents felt that they coped well with the daily injections (pediatric patients 100%; adult patients 85%; caregivers 92%). Those who did not feel they coped well (two adult patients, one family member) cited fear of injections or itching at the needle site as issues. A total of 75% of caregivers felt that their lives were significantly (42%) or partially (33%) easier after their relative started therapy, and 25% perceived little or no change in their own lives.

Representative free text responses regarding the changes observed after starting treatment are shown in Table 2; full responses are shown in Supplementary Table 3, Additional file 1. Overall, pediatric patients frequently mentioned feeling fitter and less hungry, but some also noted pigmentation changes and initial vomiting; adult patients mentioned reduced hunger and feeling more relaxed; and caregivers mentioned stabilization of body weight but also darkening of skin.

**Table 2:** Selected responses regarding changes observed after starting treatment with setmelanotide

Question	Pediatric patients	Adult patients	Caregivers
What were the changes after starting treatment?	• Feeling full, not being hungry all the time. I feel lighter. My stomach is no longer bloated and I’ve lost weight.	• The feeling of hunger has improved so that I am no longer hungry so often.	• My child felt fitter. The weight has stabilized– not gained, not lost.
	• That I wanted to do more sport. I got full sooner. That I feel fitter. That I haven’t become sad.	• No more insatiable hunger. Less grocery shopping, less cooking.	• Darkening of the skin. He eats less, doesn’t ask for seconds. And no further weight gain.
Why would you recommend the specialist nurse support service to other patients?	• Someone is always there to answer questions. You don’t feel left alone.	• Good support and instruction at the start of therapy. Being able to reach someone with questions.	• Very friendly carer. Patiently explained the individual steps to our son in his familiar home environment in a child-friendly manner. Our son looked forward to seeing the carer every day and quickly got used to the injections. We could call him at any time with questions or problems.
		• Totally great support! The initial uncertainty with the handling and the medication is reduced or taken away.	

All responses available in Additional file 1, Supplementary Table 3

### Support programs offered at the start of setmelanotide treatment

**Fig. 4:**
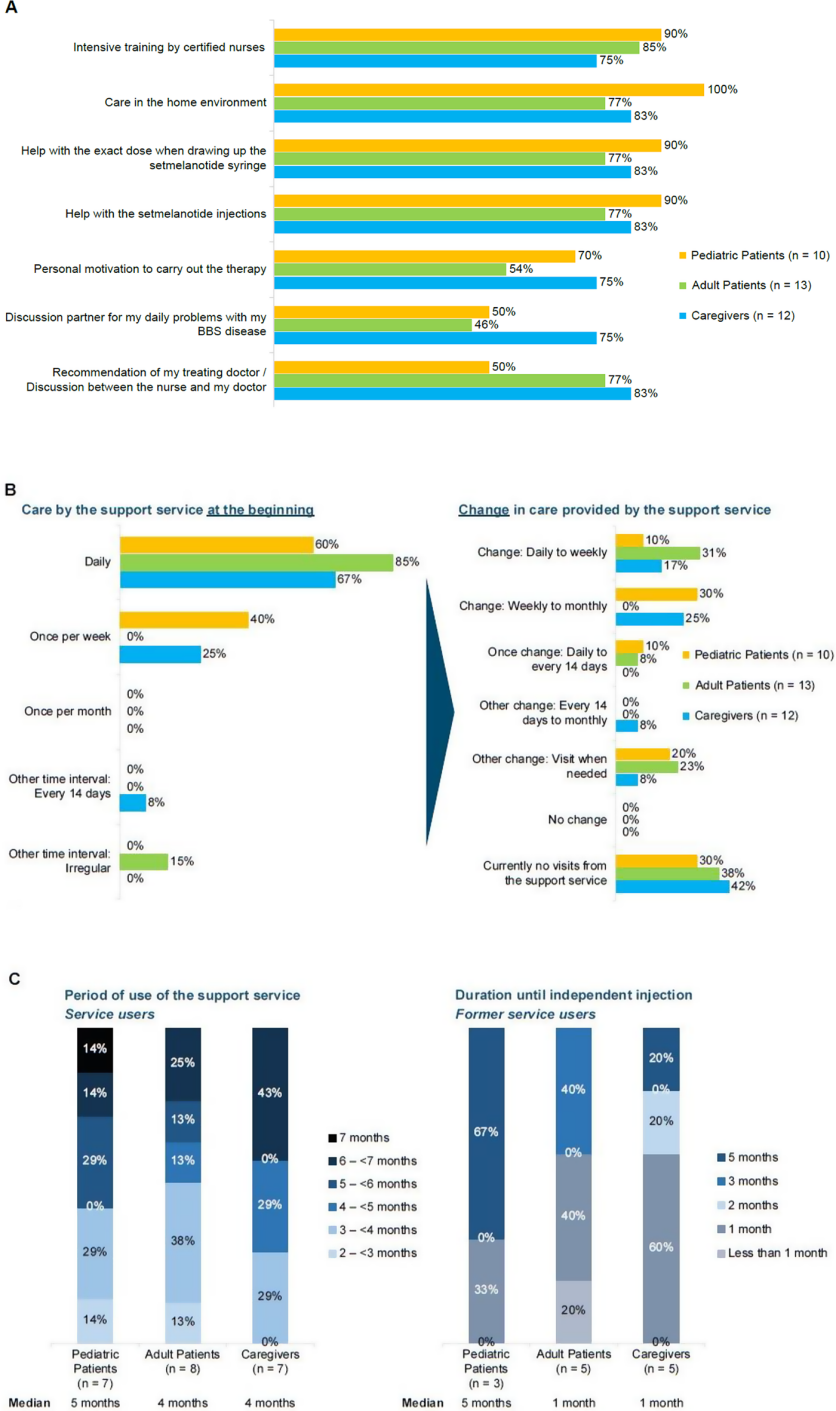
Patient and caregiver perceptions and utilization of the specialist nurse support (**A**) Nature of support provided by the specialist nurse support service. Proportion of study participants reporting 4 or 5 on a scale where 1 = “does not apply” and 5 = “applies to a high degree”. (**B**) Change in frequency of care over time by the specialist nurse support service. (**C**) Duration of care provided by the specialist nurse support service from start of treatment and at last follow-up

Few respondents used additional educational support, in addition to the specialist nurse support service, provided by a patient association (pediatric patients 10%; adult patients 15%; caregivers 8%). When asked why they chose to use the specialist nurse service, most respondents cited reasons related to home care, help with injections and dosing, access to certified nurses, and the recommendation of the treating physician (Fig. 4A).

The aim of the specialist nurse support service was to train patients and their carers to manage their care independently. At the start of therapy, support from the specialist nurse support service was provided daily or at least once per week in most cases (Fig. 4B). At last follow-up, between 30 and 42% of the respondents were no longer visited by the specialist nurse support service, or required visits only as needed (Fig. 4B).

The median duration of specialist nurse support service was approximately 5 months, although some patients have been using the service for significantly longer (Fig. 4C). The median time to independent injection was 1 month among adult patients and caregivers and was longer in pediatric patients (5 months; Fig. 4C).

The overall support package provided by the specialist nurse support service (i.e. technical instruction, professional and social expertise, overall home care) was unanimously rated as excellent by both patients and caregivers. Most patients (pediatric patients 100%; adult patients 92%) and caregivers (92%) would recommend the specialist nurse support service to others (Table 2 and Supplementary Table 3, Additional file 1), and most patients and caregivers did not express any further need for support.

## Discussion

This patient satisfaction study evaluated the experiences of patients with BBS and their caregivers during treatment with setmelanotide and the specialist nurse support to assist with their medication. The disease burden of BBS is mainly comprised of feelings of insatiable hunger, early-onset obesity and, in adult patients, loss of vision. The most dominant overall change perceived by the survey participants after starting treatment with setmelanotide was a reduction in the feeling of hunger, weight loss or no further weight gain. The overall support package provided by the specialist nurse support service was unanimously rated as excellent by both patients and caregivers.

Setmelanotide represents a crucial new option for appetite regulation and weight management in patients with BBS [17]. As documented in a recent placebo-controlled phase 3 trial, improved regulation of feelings of hunger is a key positive treatment effect in patients with BBS treated with setmelanotide [17]. Similar outcomes were observed in the current real-world study, as patients with BBS and their caregivers both reported a rapid and sustained reduction in the feeling of hunger after starting treatment with setmelanotide.

In patients with BBS, hyperphagia and weight gain are experienced early in life, which can be an overwhelming burden to patients with BBS and their caregivers [25, 26]. The burden of relentless hunger experienced by patients with BBS was reflected in both the symptoms most commonly reported prior to starting treatment with setmelanotide and by patients’ expectations of treatment. Addressing the feelings of hyperphagia is important, as many of the complications that patients with BBS experience are associated with obesity, an abnormally high level of hunger and food-seeking behavior, which can negatively affect QoL, mood, and social participation [27].,

Meaningful QoL improvements across multiple health-related QoL measures have been reported by patients with BBS treated with setmelanotide in the setting of a placebo-controlled clinical trial [11]. Although this real-world study did not evaluate QoL using validated QoL instruments, both patients with BBS and their carers reported improvements in mood, mobility, and social activities that reflect general, real-world QoL improvements after starting treatment with setmelanotide.

Fear of injection pain was a key concern amongst pediatric patients prior to starting treatment with setmelanotide. This might have contributed to the prolonged duration of specialist nurse support service in children and caregivers of children with BBS. However, most patients reported no problems with the required daily injections. Nurses play an important role in supporting both patients and caregivers, as they can share their knowledge, experience, and skill in managing injectable treatments and thus increase treatment confidence and reduce fear and anxiety, particularly in children, associated with injections [28]. The importance of supporting patients and caregivers with their daily injections was reflected by the extensive training and support provided by the specialist nurse support nurse support service, which initially provided daily or weekly support until patients or their caregivers had become sufficiently independent to administer setmelanotide. In individual cases of adult patients with BBS who live alone, the visual impairment may necessitate a personalized approach for subcutaneous medication application.

Although rare diseases are surprisingly common, with roughly 4 million people living with a rare disease in Germany, the small number of patients affected by each rare disease means that there are substantial unmet needs in the diagnosis, treatment, and support of patients living with rare diseases [29]. In Germany, patients affected by rare diseases generally report high unmet support needs across multiple aspects of care, including health information, psychological support, physical activity and activities of daily living, and sexuality needs [23]. These needs can be mitigated by additional healthcare support services, such as the specialist nurse support nurse support service assessed in this study. The high satisfaction ratings for the specialist nurse support service from both patients and caregivers reflect the added value provided by this service towards the unmet needs experienced by patients living with BBS and their families. The specialist nurse service may have contributed to improved treatment adherence and the notably low discontinuation rate (0%) observed in this study. Compared with the phase 3 study, which reported a discontinuation rate of 19% among patients with BBS over a 12-month treatment period [17], the shorter duration of this study (6 months) may also have played a role in the low discontinuation rate.

Key study limitations of this analysis include the small sample size, due primarily to the rarity of the disease, potential sample bias in the recruitment of study participants and the relatively short study period of the survey. This was a single-center study conducted at one center in Germany with extensive experience in the management of patients with BBS, which may limit the generalizability of the results. It should be noted that the survey had not been extensively validated in separate patient populations and against other instruments before it was used. Additionally, patients had a relatively short treatment duration prior to completing the survey and patient- and caregiver-reported responses were not correlated with clinical assessments and outcomes. Survey participants were asked to recall the disease symptoms present prior to starting setmelanotide treatment and so may not have been able to accurately recall their symptoms, the severity of symptoms, and their expectations of treatment. Adult patients with BBS with more severe intellectual disabilities and limited care support may be underrepresented in this study due to the limited ability to process the questionnaire. Lastly, due to a technical mistake in the questionnaire, insatiable hunger in adult patients prior to treatment start could not be reported. However, 69% of these adult patients reported a reduction in insatiable hunger.

In conclusion, this survey of pediatric and adult patients living with BBS and their caregivers demonstrated that substantial real-world improvements of key symptoms, such as relentless hunger and obesity, could be achieved through daily treatment with setmelanotide. The specialist nurse support service offering multidimensional support from the start of therapy was valued by both patients and caregivers and demonstrated how a personalized approach to healthcare support can address a high unmet need among patients living with BBS, contributing to high rates of patient satisfaction and treatment adherence.

## Electronic supplementary material

Below is the link to the electronic supplementary material.

Supplementary Material 1

Supplementary Material 2

## Data Availability

The data sets used and/or analyzed during the current study are available from the corresponding author on reasonable request.

## Abbreviations

BBS: Bardet–Biedl syndrome
MC4R: Melanocortin-4 receptor
QoL: Quality of life
