# Supplementary material for: Patient and caregiver experiences with a patient-support program for setmelanotide treatment of patients with Bardet–Biedl syndrome
Source: Orphanet J Rare Dis. 2025 Jun 8;20:290. doi: 10.1186/s13023-025-03835-9 (PMC12147271; doi:10.1186/s13023-025-03835-9)
Supplement: Supplementary file 1 — Supplementary Material 1 [file 13023_2025_3835_MOESM1_ESM.docx]

**Additional file 1**

## Supplementary Table 1. Pediatric, family member (caregiver), and adult survey questions about the specialist nurse support service [English translation]

| **Patient Questionnaire**  **(Child, duration of therapy 3-6 months)** | **Family Questionnaire**  **(only 1 person: parent, grandparent, older brother/sister)** | **Patient Questionnaire**  **(Adult, duration of therapy 3-6 months)** |
| --- | --- | --- |
| Age  Sex  Mother tongue  Ethnicity  Country of origin  Setmelanotide therapy start date | Patient:  Age  Sex  Mother tongue  Ethnicity  Country of origin  Time since BBS diagnosis  Self-rated health  Setmelanotide therapy start date | Age  Sex  Mother tongue  Ethnicity  Country of origin  Time since BBS diagnosis  Self-rated health  Setmelanotide therapy start date |
| **1. How did the following symptoms of BBS affect you before the start of the setmelanotide therapy?**  I was always hungry  I felt fat or everyone said that I was too fat  I had more fingers and toes than other children  I was afraid of the dark, I see worse in the dark  I had a hard time concentrating and I didn´t do well at school  I was often sad  I was often sick and missed school  I often argued with my sister and brother; Mom and dad often argued and scolded  I was often teased or annoyed  Others | **1. Which symptoms of BBS did you notice and how did it impair the patient?**  Insatiable hunger  Early onset obesity (overweight)  Supernumerary limbs (polydactyly)  Increasing loss of vision (night blindness, uncertainty in the dark)  Cognitive limitations (learning difficulties)  Depression and sadness  Days missed from school or absence from school, work or apprenticeship  Family burden on siblings and parents  Social exclusion/bullying  Others | **1. How did the following symptoms of BBS affect you before the start of the setmelanotide therapy?**  Insatiable hunger  Early onset obesity (overweight)  Supernumerary limbs  Increasing loss of vision (night blindness, uncertainty in the dark)  Cognitive limitations (Learning difficulties)  Depression and sadness  Days lost from work and absences  Family burden on siblings and parents  Social participation (celebrations, festivals, sport)  Others |
| **2. What expectations did you have concerning the setmelanotide therapy?**  That I will be less hungry  That I will be lose weight, getting thinner  That I will feel better overall  That I will concentrate better  That I will be less sick  That there will be fewer arguments  That I will be teased less angrily  Others | **2. What expectations did you have concerning the setmelanotide therapy?**  Reduction of insatiable hunger  Reduction of overweight  Improvement of quality of life  Improvement of liver values  Improvement of cognitive limitations  Improving the school situation  Less family conflicts  Stopping of social exclusion/bullying  Others | **2. What expectations did you have concerning the setmelanotide therapy?**  Reduction of insatiable hunger  Reduction of overweight  Improvement of quality of life  Improvement of liver values  Improvement of cognitive limitations  Improving the work situation  Less family conflicts  Stopping of social exclusion/bullying  Others |
| **3. What concerns did you have before starting setmelanotide therapy?**  That I won’t lose any weight or lose too little weight  That injection will hurt  That I will get side effects (e.g., nausea, darker skin, headaches, spontaneous erections)  That I will not cope with the injections on my own  That the preparation of the drug will be difficult  That preparing the medication and injecting will take a long time  Others | **3. What concerns did you have before your child/NN started setmelanotide therapy?**  Fear of the injection  Drug will not work or will work very slowly  Drug causes side effects (e.g., nausea, darker skin, headaches, spontaneous erections)  I will be left alone with the therapy of the patient  Preparation of the drug will be difficult  Medication will take a lot of time  Others | **3. What concerns did you have before starting setmelanotide therapy?**  Fear of the injection  Drug will not work or will work very slowly  Drug causes side effects (e.g., nausea, darker skin, headaches, spontaneous erections)  I will be left alone with therapy  Preparation of the drug will be difficult  Medication will take a lot of time  Others |
| **4. How does your daily life compare to your life without the setmelanotide therapy?**  My feeling of hunger is no longer as strong or as frequent  I have lost weight, I became thinner  I sleep better at night and stay asleep  I am full of energy and do lots of new things  I am in a good mood  I can concentrate better  I miss less school  I argue less with my brother/sister, mom and dad  I meet other children more often (e.g., birthdays, celebrations, sport)  I am more independent in my daily life  I am more mobile and can walk longer distances  Others | **4. Who is the treating doctor?**  Paediatrician  Specialist (endocrinologist, nephrologist)  Other doctors (please specify the respective doctor) | **4. Who is the treating doctor?**  General practitioner  Specialist (endocrinologist, nephrologist)  Other doctors (please specify the respective doctor) |
| **5. When did you first notice any changes?**  After a few days  After a few weeks  After a few months  Which changes did you realize? | **5. How did the life of the patient change since the start of the setmelanotide therapy?**  Reduction of insatiable hunger  Reduction of overweight  Improvement of night sleep (e.g. ability to sleep the whole night through)  Improvement of energy and performance  Improvement of cognitive limitations  Improving the work situation  Less family conflicts  Social participation (e.g. celebrations, festivals, sport)  My daily life as a relative is easier, I am more independent  The patient is more mobile and can walk longer distances  Others | **5. How does your daily life compare to your life without the setmelanotide therapy?**  Reduction of insatiable hunger  Reduction of overweight  Improvement of night sleep (e.g. ability to sleep the whole night through)  Improvement of energy and performance  Improvement of cognitive limitations  Improving the work situation  Less family conflicts  Social participation (e.g. celebrations, festivals, sport)  My daily life is easier  I am more mobile and can walk longer distances  Others |
| **6. How did your feeling of hunger change after the setmelanotide therapy?**  I no longer eat snacks between meals  I get bored sometimes because I don´t think about food all the time anymore  I am invited to parties again (birthday parties, children´s parties)  I´m full at the end of a meal  I do not seek food during the night any more  I no longer react aggressively when the food is not on the table on time  Other changes | **6. When did you first notice any changes of your child/NN?**  After a few days  After a few weeks  After a few months  Which changes did you realize? | **6. When did you first notice any changes?**  After a few days  After a few weeks  After a few months  Which changes did you realize? |
| **7. How do you currently feel about the daily injection?**  I got used to it and can handle the injection well  I still have problems with it  If there are still problems, what are they? | **7. How did your child´s feeling of hunger change after the setmelanotide therapy?**  She/he no longer eats snacks between meals  She/he gets bored sometimes because she/he doesn´t think about food all the time anymore  She/he is invited to parties again (birthday parties, children’s parties)  She/he is full at the end of a meal  She/he does not seek food during the night any more  She/he no longer reacts aggressively when the food is not on the table on time  Other changes | **7. How did your feeling of hunger change after the setmelanotide therapy?**  I no longer eat snacks between meals  I get bored sometimes because I don´t think about food all the time anymore  I am taking more part in social life again (Family celebrations, sport and culture events)  I´m full at the end of a meal  I do not seek food during the night any more  I no longer react aggressively when the food is not on the table on time  Other changes |
| **8. Which care programs were offered to you at the beginning of the therapy?**  Technical instruction (injection training)  Support of a Patient Association Group  Care by a registered nurse ()  Others | **8. How do you currently feel about the daily injection?**  I got used to it and can handle the injection well  I still have problems with it  If there are still problems, what are they? | **8. How do you currently feel about the daily injection?**  I got used to it and can handle the injection well  I still have problems with it  If there are still problems, what are they? |
| **9. What help from the specialist nurse support service is important to you?**  That I will be trained by a nurse  That I am looked after in my home environment  That I can get help with the exact dose when drawing up the setmelanotide syringe  That I am helped during my setmelanotide injections  That I am personally motivated to carry out the therapy  That I have a person to talk to about the daily problems of my BBS disease  That an exchange takes place between the nurse and my doctor  Others | **9. Which care programs were offered to you at the beginning of the therapy?**  Technical instruction (injection training)  Support of a Patient Association Group  Care by a registered nurse  Others | **9. Which care programs were offered to you at the beginning of the therapy?**  Technical instruction (injection training)  Support of a Patient Association Group  Care by a registered nurse  Others |
| **10. How often did the nurses of the specialist nurse support service visit you?**  Daily  Once per week  Once per month  Other time intervals | **10. Why did you choose the specialist nurse support service?**  Intensive training by certified nurses/carers  Care in the home environment  Help with the exact dose when drawing up the setmelanotide syringe  Help with the setmelanotide injections  Personal motivation to carry out the therapy  Discussion partner for my daily problems with my BBS disease  Recommendation of my treating doctor  Others | **10. Why did you choose the specialist nurse support service?**  Intensive training by certified nurses/carers  Care in the home environment  Help with the exact dose when drawing up the setmelanotide syringe  Help with the setmelanotide injections  Personal motivation to carry out the therapy  Discussion partner for my daily problems with my BBS disease  Recommendation of my treating doctor  Others |
| **11. Has the number of visits changed over the course of the therapy?**  From daily to weekly  From weekly to monthly  Other changes  I am currently no longer cared for by a nurse | **11. How long did you use the specialist nurse support service?**  Weeks | **11. How long did you use the specialist nurse support ervice?**  Weeks |
| **12. After what period of time were you able to do the medication on your own without the help of the specialist nurse support service ?** | **12. How often did the nurses of the specialist nurse support service visit you?**  Daily  Once per week  Once per month  Other time intervals | **12. How often did the nurses of the specialist nurse support service visit you?**  Daily  Once per week  Once per month  Other time intervals |
| **13. How do you rate the technical instruction of the specialist nurse support service concerning the injection technique?** | **13. Has the number of visits changed over the course of the therapy?**  From daily to weekly  From weekly to monthly  Other changes  I am currently no longer cared for by a nurse | **13. Has the number of visits changed over the course of the therapy?**  From daily to weekly  From weekly to monthly  Other changes  I am currently no longer cared for by a nurse |
| **14. How do you rate the professional competence of the nurses of the specialist nurse support service?** | **14. After what period of time was the patient able to do the medication on their own without the help of the specialist nurse support service ?** | **14. After what period of time were you able to do the medication on your own without the help of the specialist nurse support service ?** |
| **15. How do you rate the social competence of the nurses of the specialist nurse support service?** | **15. How do you rate the technical instruction of the specialist nurse support service concerning the injection technique?** | **15. How do you rate the technical instruction of the specialist nurse support service concerning the injection technique?** |
| **16. How do you rate the specialist nurse support service overall?** | **16. How do you rate the professional competence of the nurses of the specialist nurse support service?** | **16. How do you rate the professional competence of the nurses of the specialist nurse support service?** |
| **17. Would you recommend the specialist nurse support service to other patients?** | **17. How do you rate the social competence of the nurses of the specialist nurse support service?** | **17. How do you rate the social competence of the nurses of the specialist nurse support service?** |
|  | **18. How do you rate the specialist nurse support service overall?** | **18. How do you rate the specialist nurse support service overall?** |
|  | **19. Which additional support would you like from whom?** | **19. Which additional support would you like from whom?** |
|  | **20. Would you recommend the specialist nurse support service to other patients?** | **20. Would you recommend the specialist nurse support service to other patients?** |

## Supplementary Table 2. Affected BBS genes of patients and pathogenic variants

| **Affected BBS gene,**  **n (%)** | **Homozygous (H) /  Compound heterozygous (C)** | **Pathogenic variants** |
| --- | --- | --- |
| 1, 4 (13.8) | C | secondary: BBS 1, heterogenous, c.1396G>A; p.(Ala466Thr) |
|  | C | c.145C>T; p.(Arg49Trp) + c.680_681delinsTT; p.(Gly227Val) |
|  | H | c.985dup; p.(Thr329Asnfs*10) |
|  | H | c.1169T>G; p.(Met390Arg) |
| 2, 1 (3.4) | C | c.823C>T; p.(Arg275) + c.1986dupT; p.(Asn663) |
| 4, 1 (3.4) | H | c.(76+1_77-1)_(220+1_221-1)del (p.(Pro27_Ala74del) |
| 5, 3 (10.3) | C | c54dupC; p.(Ala19Argfs*14) + deletion Exon 10-12 |
|  | C | c54dupC; p.(Ala19Argfs*14) + deletion Exon 10-12 |
|  | C | c54dupC; p.(Ala19Argfs*14) + deletion Exon 10-12 |
| 6, 4 (13.8) | H | c.110A>G; p.(Tyr37Cys) |
|  | C | c.110A>G; p.(Tyr37Cys) + c.1436C>G; p.(Ser479*) |
|  | C | c.110A>G; p.(Tyr37Cys) + c.1436C>G; p.(Ser479*); |
|  | H | c.479+4A>G (p.?) + deletion Exon 14-17 |
| 7, 3 (10.3) | C | c.1967_1968TAdelinsC; p.(Leu656Profs*18) + deletion Exon 11 |
|  | H | c.784_793dup; p.(Asn269GlyfsX95) + c.1431_1447del; p.(Leu478ArgfsX17) |
|  | C | c.1169T>G; p.(Met390Arg) |
| 8, 3 (10.3) | H | c.776A>G; p.(Asp259Gly) |
|  | H | c.758_759delCT; p.(Ser253*) |
|  | H | deletion Exon 9 |
| 9, 1 (3.4) | H | c.263+1G>P (IVS3+1G>P) |
| 10, 6 (20.7) | H | c.1269_1273del; p.(Gln423fsX) |
|  | H | c.1269_1273del; p.(Gln423fsX) |
|  | C | c.271dupT; p.(Cys91LeufsX5) + c.273C<G; p.(Cys91Trp) |
|  | C | c.271dupT p.(Cys91LeufsX5) + c.273C>G p.(Cys91Trp) |
|  | C | c.1330dup; p.(Ser444Lysfs*6) + c.1555_1564del; p.(Thr519Argfs*2) |
|  | C | c.915delG; p.(Met305fs) |
| 12, 2 (6.9) | C | c.1483_1484delGA; p.(Glu495Argfs*3) + c.1573_1574insT; p.(Arg525Leufs*19) |
|  | H | c.1375C>T; p.(Gln459*) |
| 16, 1 (3.4) | H | c.968A>G; p.(His323Arg) |

## Supplementary Table 3. All responses from patients and family members (caregiver) [English translation]

| **Question** | **Responses** |
| --- | --- |
| What were the changes? | *Pediatric patients*   - That I wanted to do more sport. I got full sooner. That I feel fitter. That I haven't become sad. - My belly has become thinner. I can fit into nice clothes again. I've become more mobile. Fewer fights about food. My self-esteem has increased. - Moles and skin became darker, slightly blotchy. Feeling of fullness. Vomiting because I ate too much (first had to learn to recognize satiety). Performance has improved  (e.g. keeping up better on a walk without breathing heavily). - Feeling full, not being hungry all the time. I feel lighter. My stomach is no longer bloated and I've lost weight. |
|  | *Adult patients*   - I am calmer / more relaxed. My social behaviour at work has changed positively. I eat less and more consciously. - The feeling of hunger has improved so that I am no longer hungry so often. - No more insatiable hunger. Less grocery shopping, less cooking. - No more cravings for meat. |
|  | *Family members (caregivers)*   - My child felt fitter. The weight has stabilized - not gained, not lost. - Darkening of the skin. He eats less, doesn't ask for seconds. And no further weight gain. - No hunger pangs, darker skin. He is more relaxed. We receive positive feedback from relatives and friends. |
| Why would you recommend the specialist nurse support service to other patients / children? | *Pediatric patients*   - Because they are a great help and are so friendly and always there. - Because the nurses are nice and explain it well. - Very helpful, it gives you the security you need. You only have one contact person. - Someone is always there to answer questions. You don't feel left alone. - Emotional support. Someone is always there to answer questions. |
|  | *Adult patients*   - Reliable, empathetic, always ready to listen to the family. Always ready to help with problems. - Good support and instruction at the start of therapy. Being able to reach someone with questions. - Totally great support! The initial uncertainty with the handling and the medication is reduced or taken away. - Because they are very helpful and motivated and encouraged me to continue injecting. They were very nice, kind and helpful and gave me tips and tricks. THANK YOU VERY MUCH. |
|  | *Family members*   - Very good care and security. - Because the specialist nurse support service provides security in dealing with the disease and medication and strengthens confidence in one's own abilities. - Very competent professionally and socially. Comprehensible instruction in handling the medication. - Very friendly carer. Patiently explained the individual steps to our son in his familiar home environment in a child-friendly manner. Our son looked forward to seeing the carer every day and quickly got used to the injections. We could call him at any time with questions or problems. |
