# Supplementary material for: Patient and caregiver experiences with a patient-support program for setmelanotide treatment of patients with Bardet–Biedl syndrome
Source: Orphanet J Rare Dis. 2025 Jun 8;20:290. doi: 10.1186/s13023-025-03835-9 (PMC12147271; doi:10.1186/s13023-025-03835-9)

**Additional file 2**

## Supplementary Fig. 1 Symptoms commonly reported prior to starting treatment with setmelanotide

Proportion of study participants reporting “4” or “5” on a scale where “1 = does not apply” and “5 = applies to a high degree”


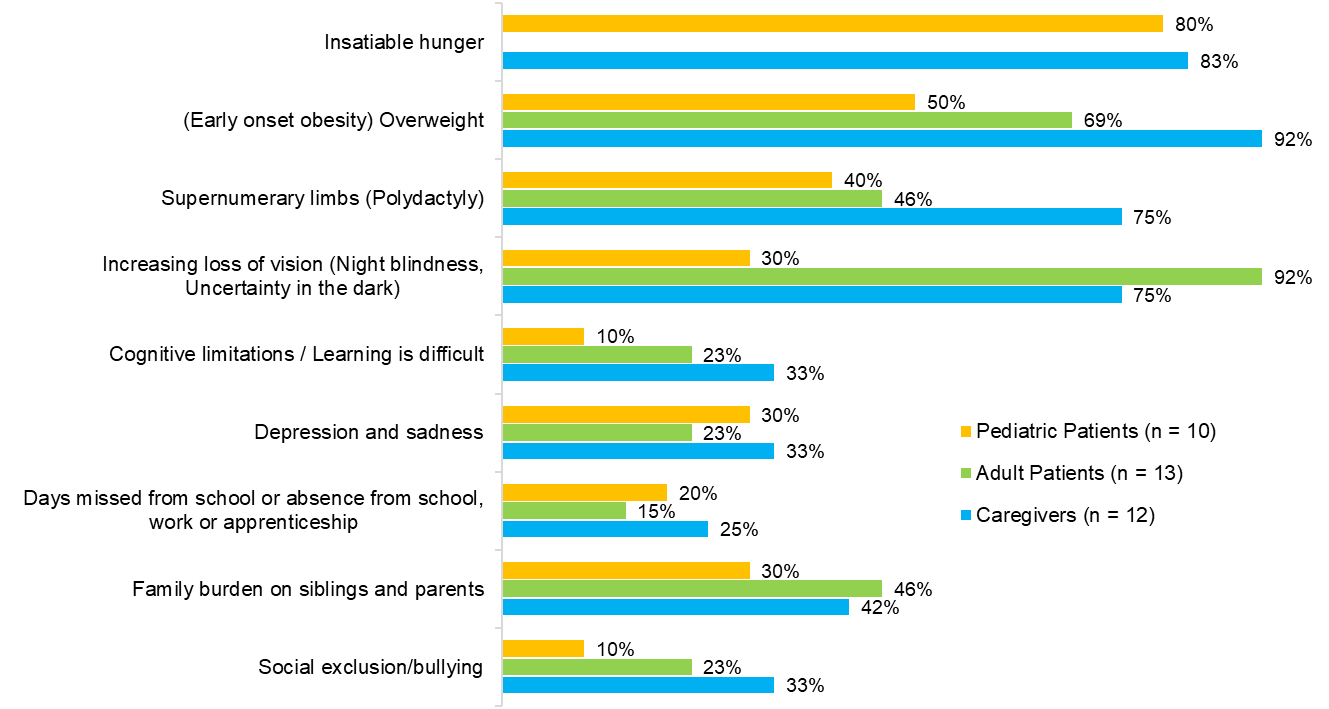


## Supplementary Fig. 2 Expectations prior to starting treatment with setmelanotide

Proportion of study participants reporting “4” or “5” on a scale where “1 = does not apply”, 2 = hardly applies; 3 = neutral; 4 = true; and “5 = applies to a high degree”


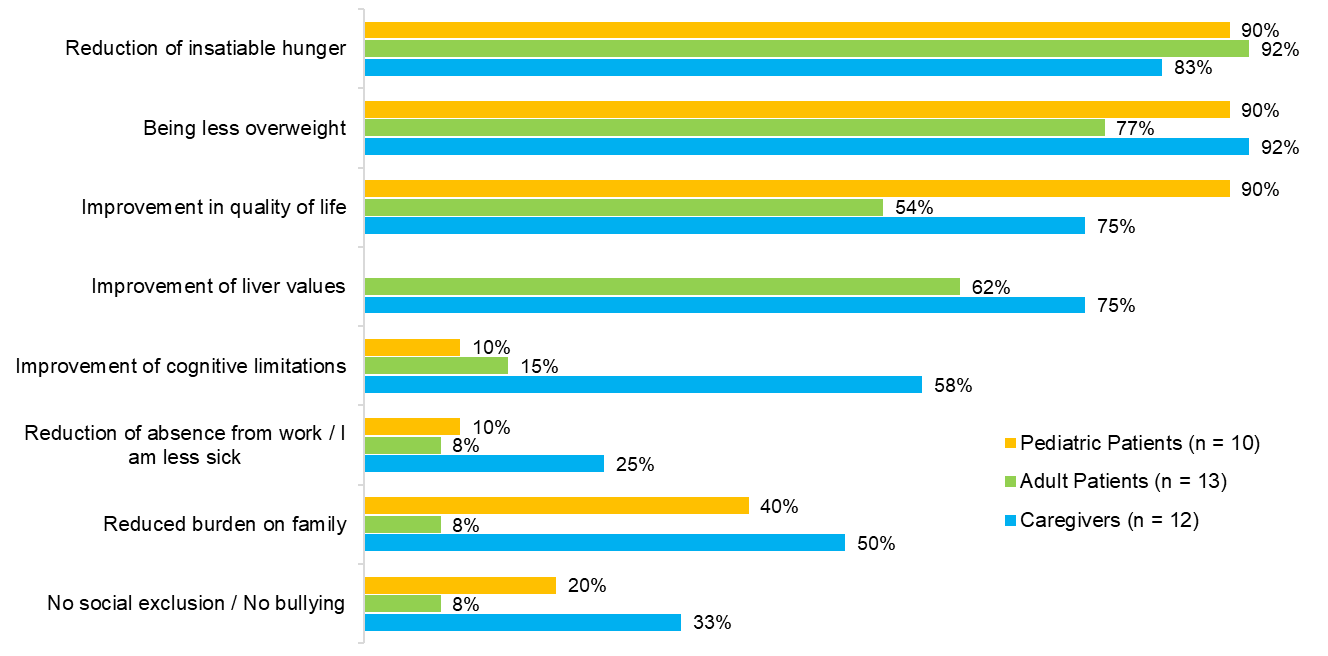


## Supplementary Fig. 3 Concerns prior to starting treatment with setmelanotide

Proportion of study participants reporting “4” or “5” on a scale where “1 = does not apply”, 2 = hardly applies; 3 = neutral; 4 = true; and “5 = applies to a high degree”


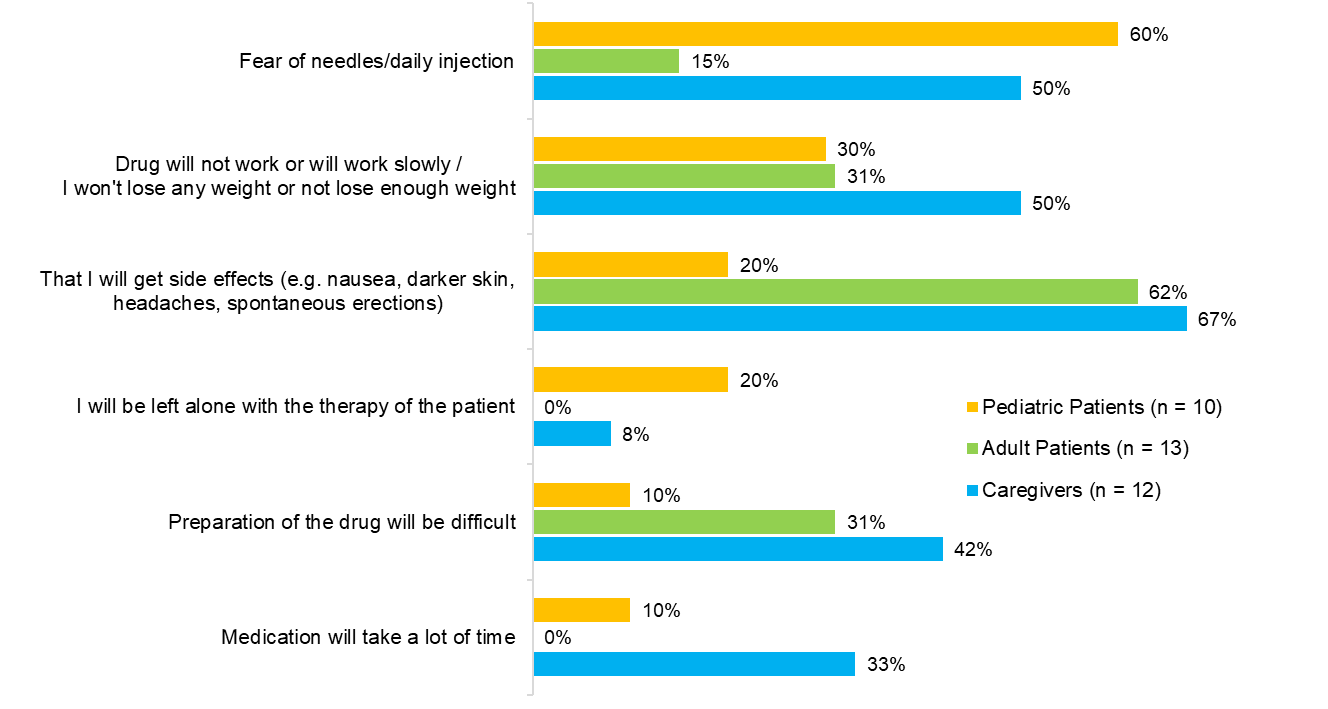

Supplement: Supplementary file 2 — Supplementary Material 2 [file 13023_2025_3835_MOESM2_ESM.docx]
